# Parathyroidectomy Is Associated with Reduced Mortality in Hemodialysis Patients with Secondary Hyperparathyroidism

**DOI:** 10.1155/2015/639587

**Published:** 2015-04-23

**Authors:** Tsung-Liang Ma, Peir-Haur Hung, Ing-Ching Jong, Chih-Yen Hiao, Yueh-Han Hsu, Pei-Chun Chiang, How-Ran Guo, Kuan-Yu Hung

**Affiliations:** ^1^Department of Internal Medicine, Ditmanson Medical Foundation Chiayi Christian Hospital, 539 Jhongsiao Road, Chiayi 600, Taiwan; ^2^Department of Applied Life Science and Health, Chia Nan University of Pharmacy and Science, No. 60, Section 1, Erren Road, Rende District, Tainan 71710, Taiwan; ^3^Department of Environmental and Occupational Health, College of Medicine, National Cheng Kung University, 1 University Road, Tainan 701, Taiwan; ^4^Department of Occupational and Environmental Medicine, National Cheng Kung University Hospital, 138 Sheng Li Road, Tainan 704, Taiwan; ^5^Department of Internal Medicine, National Taiwan University Hospital, 7 Chung Shan South Road, Zhongzheng District, Taipei City 10002, Taiwan

## Abstract

Secondary hyperparathyroidism increases morbidity and mortality in hemodialysis patients. The Kidney Disease Outcomes Quality Initiative Guidelines recommend parathyroidectomy for patients with chronic kidney disease and parathyroid hormone concentrations exceeding 800 pg/mL; however, this concentration represents an arbitrary cut-off value. The present study was conducted to identify factors influencing mortality in hemodialysis patients with parathyroid hormone concentrations exceeding 800 pg/mL and to evaluate the effects of parathyroidectomy on outcome for these patients. Two hundred twenty-one hemodialysis patients with parathyroid hormone concentrations > 800 pg/mL from July 2004 to June 2010 were identified. 21.1% of patients (*n* = 60) received parathyroidectomy and 14.9% of patients (*n* = 33) died during a mean follow-up of 36 months. Patients with parathyroidectomy were found to have lower all-cause mortality (adjusted hazard ratio [aHR]: 0.34). Other independent predictors included age ≥ 65 years (aHR: 2.11) and diabetes mellitus (aHR: 3.80). For cardiovascular mortality, parathyroidectomy was associated with lower mortality (HR = 0.31) but with a marginal statistical significance (*p* = 0.061). In multivariate analysis, diabetes was the only significant predictor (aHR: 3.14). It is concluded that, for hemodialysis patients with parathyroid hormone concentrations greater than 800 pg/mL, parathyroidectomy is associated with reduced all-cause mortality.

## 1. Introduction

Hyperparathyroidism has the potential to provoke renal osteodystrophy, hypertension, cardiomyocyte hypertrophy, insulin resistance, and calcium phosphate deposition in the vessel wall [1]. These conditions increase rates of cardiovascular events and mortality. For patients with chronic kidney disease (CKD) stage 5 who are on dialysis, the Kidney Disease: Improving Global Outcomes (KDIGO) guideline suggests maintaining intact parathyroid hormone (iPTH) values within the range of approximately two to nine times the upper normal limit [2]. A Japanese guideline suggests that the target range of iPTH is between 60 and 240 pg/mL [3]. Evidences supporting both recommendations were labeled weak in strength and low in quality (level 2C and level 2D, resp.). The PTH concentration reported to be associated with increased mortality ranges from >400 to >600 pg/mL [2] but no randomized controlled trial has yet been performed to test the proposal that achieving a specific PTH value leads to improved outcomes.

Although hyperparathyroidism can be controlled medically or surgically, severe hyperparathyroidism may increase the difficulty of medical control, especially when iPTH values exceed 800 pg/mL [4]. The Kidney Disease Outcomes Quality Initiative (K/DOQI) Clinical Practice Guidelines recommend parathyroidectomy in patients with severe hyperparathyroidism (defined as iPTH values persistently in excess of 800 pg/mL [88.0 pmol/L]) or with hypercalcemia and/or hyperphosphatemia refractory to medical therapy. The evidence supporting this recommendation has been labeled as “opinion” [5]. The Japanese guideline also recommends parathyroidectomy for severe secondary hyperparathyroidism (intact PTH levels >500 pg/mL) refractory to medical treatment (level 1B) [3]. KDIGO guidelines also recommend parathyroidectomy for patients with CKD stages 3–5D and severe hyperparathyroidism unresponsive to medical therapy. The evidence supporting this recommendation was labeled as 2B, which is defined as evidence weak in strength and moderate in quality [2]. The cut-off value of 800 pg/mL iPTH as an indication for surgery is arbitrary, and no randomized controlled studies currently exist that support the benefit of parathyroidectomy for patients with iPTH > 800 pg/mL. Furthermore, hemodialysis patients may refuse parathyroidectomy because of no remarkable symptoms, even when their iPTH values exceed 800 pg/mL. The following retrospective cohort study was therefore conducted to evaluate the relationship between parathyroidectomy and mortality in hemodialysis patients with secondary hyperparathyroidism.

## 2. Materials and Methods

### 2.1. Patients

Data for end-stage renal disease (ESRD) patients who underwent maintenance hemodialysis at Ditmanson Medical Foundation Chia-yi Christian Hospital from July 2004 to June 2010 were reviewed, and all patients with iPTH values greater than 800 pg/dL for the first time, regardless of vitamin D therapy, were enrolled. Patients were excluded if their iPTH data were unavailable or dialysis vintage was less than 3 months. Along with iPTH measurements, the data collected for each patient at the same time included age, gender, underlying diseases (including hypertension and diabetes mellitus [DM]), duration of dialysis, and Charlson comorbidity index (CCI) [6]. Data obtained from the latest monthly midweek predialysis blood tests were also collected and included albumin, hemoglobin, uric acid, calcium, phosphate, calcium × phosphate product (Ca × P product), cholesterol, and triglyceride. iPTH level was determined by a chemiluminescence immunoassay (CLIA, Immulite 2000) [7]. Single-pool *Kt*/*V* was determined using 2-point urea remodeling with the Daugirdas equation: single-pool *Kt*/*V* = −ln⁡[(1 − urea  reduction  ratio) − 0.008 × session  length] − [4 − 3.5 × (1 − urea  reduction  ratio)] × ultrafiltration/postdialysis  weight [8]. Parathyroidectomy was indicated if the patients have iPTH level greater than 800 pg/mL with failure to vitamin D therapy. Parathyroidectomy was performed if the patients accepted the operation. None of our patients received parathyroidectomy for calciphylaxis. Patients who received parathyroidectomy [9] were identified. Type of surgical intervention is judged by the surgeons responsible for parathyroidectomy. Routinely, autoimplantation of a portion of the smallest parathyroid gland to brachioradialis muscle of the non-shunt-bearing forearm was performed to prevent postoperative hypoparathyroidism. All patients were followed up from the time when the iPTH level was greater than 800 pg/mL until death or the end of the study, whichever came first. The calcimimetic cinacalcet was not used during the follow-up period.

### 2.2. Statistical Analyses

Characteristics of patients who expired were compared with those of patients who survived, and characteristics of patients who died of cardiovascular disease were compared with those of patients who survived. Cardiovascular mortality was defined as death from cerebral hemorrhage, cerebral infarction, subarachnoid hemorrhage, acute myocardial infarction, or chronic cardiac failure or as sudden death. Differences between groups were evaluated by using two-sample *t*-tests for continuous variables and Chi-square tests for categorical variables. Cox proportional hazard models were used to identify predictors of all-cause mortality and cardiovascular mortality. A backward elimination procedure was used in multivariate analyses; results are presented from a full model, which included all predictors evaluated in the study, and a final model, which included only predictors with statistical significance. In the analyses of cardiovascular mortality, patients who died from noncardiovascular causes were treated as censored, and the final model was constructed according to the final model obtained from the analyses of all-cause mortality. IBM-SPSS Version 21.0 was employed for all analyses. All statistical tests were performed at a two-sided significance level of 0.05. Informed consent was not obtained due to the retrospective design of this study and the data were analyzed anonymously. The study protocol was approved by the Institutional Review Board of the Ditmanson Medical Foundation Chia-yi Christian Hospital.

## 3. Results

Two hundred twenty-one hemodialysis patients were identified who had iPTH values >800 pg/mL during the study period. The mean age at the beginning of follow-up was 59.0 years. During a mean follow-up of 36 months (range, 7–313 months; mean deviation 21.3 months), 27.1% of patients (*n* = 60) underwent parathyroidectomy. For these 60 patients, parathyroid hormone concentrations decreased from a mean preoperative iPTH value of 2357 pg/mL (range, 819–4697 pg/mL) to a mean postoperative iPTH value of 128 pg/mL (range, 1.3–967 pg/mL). There were 3 patients with postparathyroidectomy level of iPTH >800 pg/mL and no intervention was performed for them. Overall, 33 patients expired, yielding an all-cause mortality rate of 14.9%. Of these 33 patients, 21 died of cardiovascular disease. The mortality rate for patients who received parathyroidectomy (6.7%) was lower than that for patients who did not receive parathyroidectomy (18.0%). No perioperative death was noted. Aluminum values for all patients in the study were lower than 50 *μ*g/L, values well below those were considered by the K/DOQI guidelines to be toxic to ESRD patients (60 *μ*g/L).

As compared to survivors, patients who expired were older, had a higher prevalence of DM, had higher CCI measurements, and had a lower prevalence of hyperuricemia; additionally, a smaller proportion of those who expired had received parathyroidectomy (Table 1). The mean baseline iPTH value for survivors was 1133.76 pg/mL and for nonsurvivors was 1158.42 pg/mL.

The findings from Cox proportional hazard regression analyses for all-cause mortality are shown in Table 2. Univariate analysis revealed that the risk factors included age ≥65 years (hazard ratio [HR] = 3.31), DM (HR = 4.61), and higher CCI values (CCI ≥ 5; HR = 3.30). By contrast, patients with hyperuricemia and patients who had received parathyroidectomy had lower all-cause mortality (HR values of 0.43 and 0.25, resp.). Multivariate analysis revealed that age ≥65 years (aHR = 2.11, 95% confidence interval [CI]: 1.01–4.38) and DM (aHR = 3.80, 95% CI: 1.73–8.37) were significant independent risk factors whereas parathyroidectomy was a significant independent protective factor (aHR = 0.34, 95% CI: 0.12–0.99).

For cardiovascular mortality, univariate analyses confirmed that age ≥65 (HR = 3.64), DM (HR = 3.94), and CCI > 5 (HR = 2.99) were significant risk factors whereas parathyroidectomy was associated with a lower mortality (HR = 0.31) with marginal statistical significance (*p* = 0.061) (Table 3). Multivariate analysis also revealed that DM was the only significant predictor in the final model (aHR = 3.14, 95% CI: 1.19–8.29) whereas the decrease in mortality associated with parathyroidectomy (aHR = 0.44) again failed to reach statistical significance (95% CI: 0.13–1.57). In the final model, age ≥65 years (aHR = 2.46, 95% CI: 0.98–6.19) reached marginal statistical significance as a predictor (*p* = 0.056).

**Figure 1:**
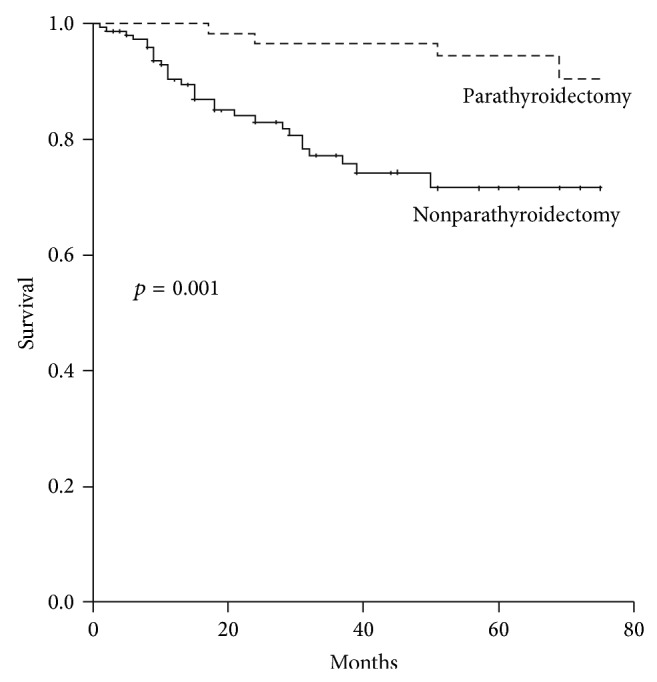
Kaplan-Meier survival curve for all-cause mortality in patients who received parathyroidectomy and patients who did not receive parathyroidectomy.

Figure 1 showed the Kaplan-Meier survival curve demonstrating that patients who had received parathyroidectomy had lower all-cause mortality (*p* = 0.001 for log-rank test).

## 4. Discussion

The most important finding of the present study is that parathyroidectomy is associated with reduced all-cause mortality for hemodialysis patients with iPTH concentrations exceeding 800 pg/mL.

Although parathyroidectomy was also associated with reduced cardiovascular mortality in these patients, statistical significance was not achieved. Because the reduction in cardiovascular mortality was relatively large (HR = 0.31 in the univariate analysis and aHR = 0.44 in the final model) and did not change significantly after adjusting for other factors, it is likely that the inability to reach statistical significance was attributable to the relatively small number of cases of cardiovascular mortality (21 patients) in the study period. Nonetheless, a PubMed search using “hemodialysis” and “hyperparathyroidism” as search terms failed to identify any study involving larger numbers of hemodialysis patients with iPTH concentrations exceeding 800 pg/mL.

Four epidemiological studies have been performed to investigate the relationship between parathyroidectomy and the survival of hemodialysis patients with hyperparathyroidism, with inconsistent findings obtained. In the prospective cohort study by Costa-Hong et al. [10] involving 118 hemodialysis patients with severe hyperparathyroidism unresponsive to medical treatment, 50 patients received parathyroidectomy and had lower all-cause mortality. In a retrospective study performed by Trombetti et al. [11], 40 ESRD patients who received parathyroidectomy for secondary hyperparathyroidism were compared with 80 matched control patients; after adjustments for age and comorbidities, no significant effects on survival were observed. These investigators suspected that the patients who had received parathyroidectomy represented a subset of healthier patients. In another retrospective cohort study [12] comparing 150 dialysis patients who underwent near-total parathyroidectomy for secondary hyperparathyroidism to matched controls from the United States Renal Data System (USRDS) database, better all-cause and cardiovascular survival were observed for the parathyroidectomy group but iPTH values for the control group were unavailable. A potential confounding factor for this study was the lack of explanation as to why patients in the control group did not receive parathyroidectomy. The most recently published study [13] retrospectively compared 88 chronic dialysis patients who received total parathyroidectomy (iPTH values > 500 pg/mL with resistance to vitamin D receptor agonists) to 88 matched controls who did not receive parathyroidectomy. After 4.41 years of follow-up, parathyroidectomy was found to be associated with higher survival rates (90.4% versus 67.4%). A recently published article by Komaba et al. compared patients with secondary hyperparathyroidism treated by parathyroidectomy with propensity score-matched control group. They found that, compared to the matched controls, patients who had undergone parathyroidectomy had a 34% and 41% lower risk for all-cause and cardiovascular mortality, respectively [14].

In two of the four studies described above, a reduction in cardiovascular mortality was observed for patients who had received parathyroidectomy, a 33% reduction in cardiovascular mortality was observed in one study [12], and a higher cardiovascular death-free survival rate (94.6% versus 76.3%) was observed in the other [13]. In addition, Costa-Hong et al. [10] reported an association of parathyroidectomy with a reduced incidence of major cardiovascular events. The small number of cases of cardiovascular mortality and, consequently, the limited statistical power, rather than inefficacy, is proposed to explain the lack of statistical significance regarding the association between reduced cardiovascular mortality and parathyroidectomy in the present study.

The cardiovascular complications of DM contribute to the higher mortalities observed for diabetic patients [15]. Of the patients in the present study, 44% had DM, a higher percentage than those reported in other studies (range of 9% [12] to 22% [13]). In the present study, DM was found to be a risk factor contributing to higher all-cause and cardiovascular mortality. Based on the findings of a recent cohort study [16], it was concluded that parathyroidectomy may reduce cardiovascular events in nondiabetic hemodialysis patients with secondary hyperparathyroidism; however, the possibility that parathyroidectomy reduces cardiovascular mortality on a long-term basis was not evaluated. Findings of the present study reveal that parathyroidectomy reduces all-cause mortality independently of DM. A similar effect of the surgery on cardiovascular mortality was observed although the reduction did not reach statistical significance.

Limitations of the present study should be addressed. First, although the number of hemodialysis patients with iPTH values exceeding 800 pg/mL was larger than that in any previously published study, the number of deaths was relatively small. Second, patients who had received parathyroidectomy could have been relatively healthier at baseline and, therefore, better candidates for surgery; full adjustment for this potential confounder was not possible. Nonetheless, aHR values obtained from the full models were very similar to those obtained from the final models, supporting relatively small confounding effects. Fourth, the duration of hyperparathyroidism before parathyroidectomy might have an effect on the outcome. Because this is a retrospective study and the date of the parathyroidectomy cannot be detected in the patients' medical records, time-dependent analysis could not be done. Furthermore, the length of comorbidities, medications such as vitamin D analogs and calcimimetic agents, and adherence to medical regimens were potential confounding factors. These factors, however, were unavailable in our medical records. More well-designed prospective studies, especially with propensity matched control, are needed to solve these limitations.

In conclusion, parathyroidectomy serves to lower all-cause mortality in hemodialysis patients with secondary hyperparathyroidism with iPTH concentrations in excess of 800 pg/mL. Although the reduction in cardiovascular mortality did not reach statistical significance, further prospective studies with larger case numbers are needed to confirm this finding. The findings of this study strongly support the benefits of parathyroidectomy for hemodialysis patients with marked secondary hyperparathyroidism and poor responses to medical therapy. It is recommended that all ESRD patients with iPTH values > 800 pg/mL receive parathyroidectomy unless contraindications exist.

## Figures and Tables

**Table 1:** Comparison of baseline characteristics of survivors and nonsurvivors from 221 hemodialysis patients with iPTH over 800 pg/mL.

			Survival status		*p* value
			Survival	Death	
			(*n* = 188)	(*n* = 33)	
Basic characteristics					

Age (year)	<65	*n *	131	14	0.002^∗^
		%	69.7%	42.4%	
	≥65	*n *	57	19	
		%	30.3%	57.6%	
	Mean		57.5 ± 12.9	67.6 ± 11.0	<0.001^∗∗^

Sex	Female	*n *	113	16	0.212^∗^
		%	60.1%	48.5%	
	Male	*n *	75	17	
		%	39.9%	51.5%	

Hypertension	No	*n *	68	15	0.310^∗^
		%	36.2%	45.5%	
	Yes	*n *	120	18	
		%	63.8%	54.5%	

Diabetes mellitus	No	*n *	114	9	<0.001^∗^
		%	60.6%	27.3%	
	Yes	*n *	74	24	
		%	39.4%	72.7%	

Duration of dialysis (month)	Mean		97.6 ± 54.4	84.9 ± 56.0	0.221^∗∗^

*Kt*/*V*	Mean		1.38 ± 0.24	1.40 ± 0.20	0.716^∗∗^

Biochemical parameters					

Hemoglobin (g/dL)	<10	*n *	82	16	0.604^∗^
		%	43.6%	48.5%	
	≥10	*n *	106	17	
		%	56.4%	51.5%	
	Mean		10.2 ± 1.4	10.3 ± 1.7	0.645^∗∗^

Albumin (g/dL)	<3.5	*n *	6	3	0.135^∗^
		%	3.2%	9.1%	
	≥3.5	*n *	182	30	
		%	96.8%	90.9%	
	Mean		4.12 ± 0.30	3.94 ± 0.36	0.003^∗∗^

Hyperuricemia (mg/dL)	<7.2	*n *	18	8	0.034^∗^
		%	9.6%	24.2%	
	≥7.2	*n *	170	25	
		%	90.4%	75.8%	
	Mean		8.19 ± 1.60	7.53 ± 2.05	0.039^∗∗^

Phosphate (mg/dL)	<5.5	*n *	55	10	0.903^∗^
		%	29.3%	30.3%	
	≥5.5	*n *	133	23	
		%	70.7%	69.7%	
	Mean		6.48 ± 1.62	5.78 ± 1.45	0.022^∗∗^

Ca × P ([mg/dL]^2^)	≥55	*n *	120	20	0.723^∗^
		%	63.8%	60.6%	
	<55	*n *	68	13	
		%	36.2%	39.4%	
	Mean		62.28 ± 16.64	54.50 ± 14.03	0.006^∗∗^

Cholesterol (mg/dL)	<200	*n *	120	22	0.754^∗^
		%	63.8%	66.7%	
	≥200	*n *	68	11	
		%	36.2%	33.3%	
	Mean		188.9 ± 46.2	178.3 ± 32.0	0.208^∗∗^

Triglyceride (mg/dL)	<150	*n *	96	20	0.311^∗^
		%	51.1%	60.6%	
	≥150	*n *	92	13	
		%	48.9%	39.4%	
	Mean		189.8 ± 190.4	152.0 ± 91.5	0.265^∗∗^

iPTH (pg/mL)	<2000	*n *	181	31	0.626^∗^
		%	96.3%	93.9%	
	≥2000	*n *	7	2	
		%	3.7%	6.1%	
	Mean		1133.8 ± 391.6	1158.4 ± 372.8	0.730^∗∗^

CCI	<5	*n *	108	9	0.001^∗^
		%	57.4%	27.3%	
	≥5	*n *	80	24	
		%	42.6%	72.7%	
	Mean		4.37 ± 1.88	5.52 ± 2.06	0.002^∗∗^

Intervention					

Parathyroidectomy	No	*n *	132	29	0.035^∗^
		%	70.2%	87.9%	
	Yes	*n *	56	4	
		%	29.8%	12.1%	

CCI: Charlson comorbidity index.

^∗^Chi-square test.

^∗∗^ *t* test.

**Table 2:** Cox proportional hazard regression of overall survival analysis for 221 hemodialysis patients with iPTH over 800 pg/mL.

	Univariate regression model				Multivariate regression model (*n* = 218)							
					Full model				Final model			
	95.0% CI for HR				95.0% CI for aHR				95.0% CI for aHR			
	HR	Lower	Upper	*p* value	aHR	Lower	Upper	*p* value	aHR	Lower	Upper	*p* value
Age ≥65	3.31	1.63	6.72	0.001	2.43	1.08	5.50	0.033	2.11	1.01	4.38	0.046
Male gender	1.52	0.77	3.02	0.228	1.21	0.54	2.73	0.646				
Hypertension	0.74	0.37	1.47	0.389	0.71	0.32	1.56	0.391				
Diabetes mellitus	4.61	2.13	10.00	<0.001	2.74	1.17	6.40	0.020	3.80	1.73	8.37	0.001
Hb >10 g/dL	0.77	0.39	1.53	0.455	0.37	0.15	0.92	0.032				
Albumin ≤3.5 g/dL	1.56	0.46	5.28	0.473	0.49	0.10	2.33	0.371				
Hyperuricemia	0.43	0.19	0.95	0.038	0.47	0.19	1.17	0.104				
Phosphate <5.5 mg/dL	1.22	0.58	2.57	0.595								
Ca × P <55 (mg/dL)^2^	1.56	0.77	3.14	0.217	1.11	0.50	2.44	0.799				
Cholesterol ≥200 m/dL	0.87	0.42	1.80	0.699	1.34	0.52	3.48	0.546				
Triglyceride ≥150 mg/dL	0.81	0.40	1.63	0.556	0.85	0.34	2.16	0.733				
PTH ≥1500 pg/mL	0.89	0.31	2.53	0.825								
PTH ≥2000 pg/mL	1.44	0.35	6.05	0.616	1.95	0.35	10.80	0.443				
CCI ≥5	3.30	1.53	7.12	0.002	2.22	0.97	5.09	0.060				
Parathyroidectomy	0.25	0.09	0.73	0.011	0.35	0.11	1.11	0.075	0.34	0.12	0.99	0.047

**Table 3:** Cox proportional hazard regression of cardiovascular survival analysis for 221 hemodialysis patients with iPTH over 800 pg/mL.

	Univariate regression model					Multivariate regression model (*n* = 203)						
						Full model			Final model			
	95.0% CI for HR					95.0% CI for aHR			95.0% CI for aHR			
	HR	Lower	Upper	*p* value	aHR	Lower	Upper	*p* value	aHR	Lower	Upper	*p* value
Age ≥65	3.64	1.51	8.80	0.004	3.21	1.15	8.94	0.026	2.46	0.98	6.19	0.056
Male gender	1.06	0.44	2.51	0.904	0.87	0.32	2.38	0.789				
Hypertension	0.80	0.34	1.89	0.606	0.71	0.27	1.87	0.486				
Diabetes mellitus	3.94	1.52	10.21	0.005	2.62	0.95	7.27	0.064	3.14	1.19	8.29	0.021
Hb >10 g/dL	0.95	0.40	2.25	0.904	0.52	0.18	1.47	0.218				
Albumin ≤3.5 g/dL	0.98	0.13	7.29	0.982	0.35	0.03	3.73	0.384				
Hyperuricemia	0.62	0.21	1.83	0.381	0.68	0.20	2.33	0.542				
Phosphate <5.5 mg/dL	0.66	0.22	1.95	0.449								
Ca × P <55 (mg/dL)^2^	1.16	0.47	2.87	0.756	0.78	0.29	2.08	0.619				
Cholesterol ≥200 m/dL	1.19	0.50	2.84	0.687	1.40	0.47	4.16	0.546				
Triglyceride ≥150 mg/dL	1.07	0.45	2.53	0.873	0.83	0.28	2.51	0.746				
PTH ≥1500 pg/mL	0.65	0.15	2.79	0.563								
PTH ≥2000 pg/mL	2.21	0.51	9.51	0.286	2.81	0.46	17.23	0.265				
CCI ≥5	2.99	1.16	7.72	0.024	2.15	0.78	5.89	0.138				
Parathyroidectomy	0.31	0.09	1.06	0.061	0.43	0.11	1.67	0.221	0.44	0.13	1.57	0.208

## References

[1] Hörl W. H. (2004). The clinical consequences of secondary hyperparathyroidism: focus on clinical outcomes. *Nephrology Dialysis Transplantation*.

[2] Kidney Disease: Improving Global Outcomes (KDIGO) CKD-MBD Work Group (2009). KDIGO clinical practice guideline for the diagnosis, evaluation, prevention, and treatment of chronic kidney disease–mineral and bone disorder (CKD–MBD). *Kidney International*.

[3] Fukagawa M., Yokoyama K., Koiwa F. (2013). Clinical practice guideline for the management of chronic kidney disease-mineral and bone disorder. *Therapeutic Apheresis and Dialysis*.

[4] National Kidney Foundation (2003). K/DOQI clinical practice guidelines for bone metabolism and disease in chronic kidney disease. *American Journal of Kidney Disease*.

[5] Moe S. M., Chertow G. M., Coburn J. W. (2005). Achieving NKF-K/DOQI bone metabolism and disease treatment goals with cinacalcet HCl. *Kidney International*.

[6] Charlson M. E., Pompei P., Ales K. L., MacKenzie C. R. (1987). A new method of classifying prognostic comorbidity in longitudinal studies: development and validation. *Journal of Chronic Diseases*.

[7] Amal L., Bergmann P. (2004). Evaluation of a chemiluminescence immunoassay for the determination of intact parathyroid hormone using the ADVIA Centaur. *Clinical Laboratory*.

[8] Daugirdas J. T. (1993). Second generation logarithmic estimates of single-pool variable volume Kt/V: An analysis of error. *Journal of the American Society of Nephrology*.

[9] Schneider R., Slater E. P., Karakas E., Bartsch D. K., Schlosser K. (2012). Initial parathyroid surgery in 606 patients with renal hyperparathyroidism. *World Journal of Surgery*.

[10] Costa-Hong V., Jorgetti V., Gowdak L. H. W., Moyses R. M. A., Krieger E. M., De Lima J. J. G. (2007). Parathyroidectomy reduces cardiovascular events and mortality in renal hyperparathyroidism. *Surgery*.

[11] Trombetti A., Stoermann C., Robert J. H. (2007). Survival after parathyroidectomy in patients with end-stage renal disease and severe hyperparathyroidism. *World Journal of Surgery*.

[12] Sharma J., Raggi P., Kutner N. (2012). Improved long-term survival of dialysis patients after near-total parathyroidectomy. *Journal of the American College of Surgeons*.

[13] Iwamoto N., Sato N., Nishida M. (2012). Total parathyroidectomy improves survival of hemodialysis patients with secondary hyperparathyroidism. *Journal of Nephrology*.

[14] Komaba H., Taniguchi M., Wada A., Iseki K., Tsubakihara Y., Fukagawa M. (2015). Parathyroidectomy and survival among Japanese hemodialysis patients with secondary hyperparathyroidism. *Kidney International*.

[15] Karamé A., Labeeuw M., Trolliet P. (2009). The impact of type 2 diabetes on mortality in end-stage renal disease patients differs between genders. *Nephron: Clinical Practice*.

[16] Lin H. C., Chen C. L., Lin H. S. (2014). Parathyroidectomy improves cardiovascular outcome in nondiabetic dialysis patients with secondary hyperparathyroidism. *Clinical Endocrinology*.

